# Functional Activity of Human Induced Pluripotent Stem Cell-Derived Cardiomyocytes on a Mouse Renal Subcapsular Xenograft Model

**DOI:** 10.3390/ijms24129792

**Published:** 2023-06-06

**Authors:** Elena V. Chepeleva, Sophia V. Pavlova, Nataliya P. Bgatova, Alexander M. Volkov, Galina M. Kazanskaya, David S. Sergeevichev

**Affiliations:** 1 Federal State Budgetary Institution National Medical Research Center Named after Academician E.N. Meshalkin of the Ministry of Health of the Russian Federation, 15, Rechkunovskaya Str., 630055 Novosibirsk, Russia; 2 Research Institute of Clinical and Experimental Lymphology–Branch of the Institute of Cytology and Genetics Siberian Branch of Russian Academy of Sciences, 2, Timakova Str., 630060 Novosibirsk, Russia; 3 Federal Research Center Institute of Cytology and Genetics Siberian Branch of Russian Academy of Sciences, 10, Ac. Lavrentiev Ave., 630090 Novosibirsk, Russia; 4 Institute of Molecular Biology and Biophysics–Subdivision of FRC FTM, 2/12, Timakova Str., 630060 Novosibirsk, Russia

**Keywords:** cardiomyocytes, induced pluripotent stem cells, cell therapy, calcium imaging, fluorescence microscopy, transmission electron microscopy

## Abstract

In the treatment of coronary heart disease, the most promising approach for replacing lost contractile elements involves obtaining cardiomyocytes through cardiac differentiation of pluripotent cells. The objective of this study is to develop a technology for creating a functional layer of cardiomyocytes derived from iPSCs, capable of generating rhythmic activity and synchronous contractions. To expedite the maturation of cardiomyocytes, a renal subcapsular transplantation model was employed in SCID mice. Following explantation, the formation of the cardiomyocyte contractile apparatus was assessed using fluorescence and electron microscopy, while the cytoplasmic oscillation of calcium ions was evaluated through visualization using the fluorescent calcium binding dye Fluo-8. The results demonstrate that transplanted human iPSC-derived cardiomyocyte cell layers, placed under the fibrous capsules of SCID mouse kidneys (for up to 6 weeks), initiate the development of an organized contractile apparatus and retain functional activity along with the ability to generate calcium ion oscillations even after removal from the body.

## 1. Introduction

Coronary heart disease (CHD), also known as ischemic heart disease, is a prevalent condition that stands as the leading cause of death and disability among adults [1]. While surgical and pharmacological advancements have improved CHD treatments and increased patient survival rates, individuals who have experienced a myocardial infarction still face a substantial risk of post-infarction complications and a decline in their quality of life [2]. Due to the heart’s limited capacity to metabolize ischemia and hypoxia in cardiac cells, a cascade of unfavorable events occurs, including apoptosis, necrosis, inflammatory reactions, remodeling, scar formation, and ultimately, the progression to end-stage heart failure [3]. Consequently, the development and implementation of new methods to restore blood flow in ischemic myocardial areas have remained an urgent challenge in cardiac surgery for many years [4]. The exploration of alternative approaches for myocardial revascularization, such as tissue engineering and cellular technology, has garnered significant attention in the treatment of CHD.

There are two primary approaches in the development of cell therapy methods for treating CHD. The first approach involves cell transplantation to replace the lost contractile elements of the myocardium, while the second approach focuses on cell transplantation to stimulate regenerative processes and restore blood supply in ischemically affected tissues [4,5]. One method of cell replacement therapy involves the transplantation of iPSC-derived cardiomyocytes to restore lost myocardial tissue [6,7]. These differentiated cardiomyocytes are not only contractile and excitable but also responsive to signals from the autonomic nervous system [8]. Furthermore, iPSC-derived cardiomyocytes serve as an abundant source of human cardiac cells for cell-based therapies, particularly for patients with severe forms of CHD [9]. However, they exhibit functional immaturity and display a fetal phenotype [6]. In contrast, postnatal cardiomyocytes possess longer and more organized sarcomere structures than fetal and iPSC-derived cardiomyocytes, enabling rapid excitation–contraction coupling and proper force generation [10]. Ideally, for myocardial repair, iPSC-derived cardiomyocytes should exhibit electrical and mechanical properties that closely resemble those of native myocardium [11]. It is anticipated that such mature cells would exhibit enhanced contractile performance and the ability to release calcium from the sarcoplasmic reticulum in synchrony, thus enabling the myocardium to function as a syncytium [12].

Therefore, it is crucial to investigate the dynamics of action potential formation and calcium currents during the maturation of cardiomyocytes derived from iPSCs. Additionally, assessing the functional status of cardiomyocytes after transplantation is essential for the development of approaches in cell replacement therapy for CHD. Various changes occur during the maturation of cardiomyocytes, including the formation of the contractile apparatus and sarcoplasmic reticulum structures, stabilization of intracellular calcium ion oscillation, production of mature isoforms of ion channels and connexins, and an increase in the number of mitochondria. Achieving the adult form of human cardiomyocytes in terms of size, shape, molecular composition, metabolism, and physiological function in vivo requires several years [11]. To expedite these processes, techniques such as cell layer formation, cultivation under electric current stimulation, mechanical stretching, and in vivo persistence are employed [6,7,13,14,15,16]. However, although significant progress has been made in the maturation of iPSC-derived cardiomyocytes, they still do not fully replicate the phenotype of adult cells. Furthermore, there is currently no universally accepted standard for defining the acceptable level of maturation of iPSC-derived cardiomyocytes for specific applications [3,12]. Further research is necessary to identify the key processes in cell development that synergistically contribute to the maturation of iPSC-derived cardiomyocytes. Striking a balance between leveraging natural developmental cues and mimicking the developing microenvironment through tissue engineering techniques can likely enhance the maturation of iPSC-derived cardiomyocytes more effectively [12].

The objective of this study was to develop a technique for creating functional layers of human iPSC-derived cardiomyocytes capable of generating rhythmic and synchronous contractions. Additionally, the study aimed to investigate the potential for accelerating cardiomyocyte maturation through subcapsular renal transplantation into SCID mice.

## 2. Results and Discussion

**Figure 1:**
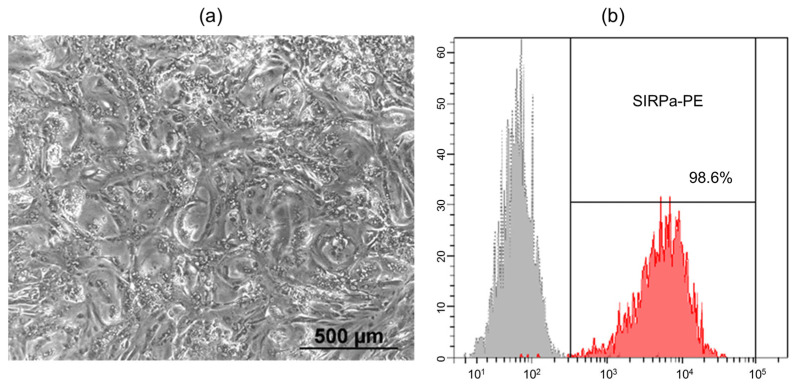
(**a**) The morphology of the human iPSC-derived cardiomyocytes on day 20 of differentiation (phase-contrast microscopy, bar 500 µm); (**b**) Expression of SIRPa on human iPSC-derived cardiomyocytes after metabolic selection (flow cytometry), grey–isotype control, red–stained cells.

The human induced pluripotent stem cell line (iMA-1L) used in this study was derived from embryonic fibroblasts of the MAN1 line at the Laboratory of Epigenetics of Development, ICG SB RAS [17]. The differentiation protocol employed a WNT signaling pathway activation to generate mesodermal precursors, followed by terminal differentiation into cardiomyocytes with subsequent inhibition of the WNT pathway [18,19]. Spontaneously contracting areas were observed on days 8–10 of differentiation, and their number and intensity increased over time. When cultured as a complete flat monolayer, the iPSC-derived cardiomyocytes exhibited a uniform distribution and acquired a rounded morphology (Figure 1a). To remove undifferentiated cells from the culture, metabolic selection was performed using a medium containing lactate instead of glucose. Lactate is exclusively metabolized by cardiomyocytes, leading to the elimination of other cell types in the absence of glucose. Flow cytometry analysis using SIRPa (CD172a) antibodies, a marker of iPSC-derived cardiomyocytes, showed that after metabolic selection, the differentiated cell population consisted of 98.6% cardiomyocytes (Figure 1b) [20].

**Figure 2:**
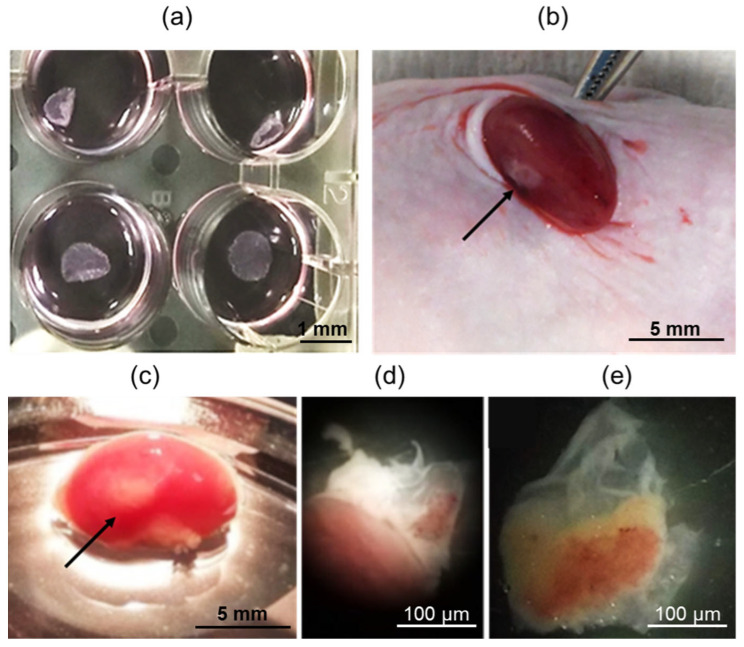
(**a**) iPSC-derived cardiomyocyte cell layers on heat-sensitive plastic; (**b**) Introducing a layer of human iPSC-derived cardiomyocytes under the SCID mouse kidney capsule, arrow–iPSC-derived cardiomyocyte cell layer on the kidney surface; (**c**) General view of the mouse kidney 42 days after iPSC-derived cardiomyocyte cell layer subcapsular transplantation, arrow–iPSC-derived cardiomyocyte cell layer on the kidney surface; (**d,e**) Transplants dissected from the kidney fibrous capsule. Bar 1 mm (**a**), 5 mm (**b**,**c**), 100 µm (**d**,**e**).

In the next step, spontaneously contracting iPSC-derived cardiomyocytes were seeded onto heat-sensitive plastic at a density of 250,000 cells per 1 cm^2^. The resulting functional cell layers were detached from the plastic at room temperature, as depicted in Figure 2a. These cell layers were then injected beneath the kidney capsule of SCID mice, as shown in Figure 2b. SCID mice are known for their severe combined immunodeficiency syndrome caused by mutations in the RAG genes, which are responsible for the rearrangement of immunoglobulin and T-cell receptor genes. Due to the reduced rejection response of xenografts in SCID mice, they have been widely utilized as a model for transplanting tissue samples from other species, including humans [21,22]. The kidney is particularly suitable for such transplants, given its easy accessibility and the ability to contain transplanted tissues under the renal capsule in a highly vascularized environment [23,24,25].

The mice showed a smooth recovery without any complications, exhibiting no signs of discomfort or infection throughout the duration of the experiment. After 42 days, when the animals were euthanized, all transplants were easily identifiable and found to be localized effectively under the renal capsule, as depicted in Figure 2c. The transplants were then carefully dissected away from the fibrous capsule and the kidney body, as shown in Figure 2d,e, respectively.

**Figure 3:**
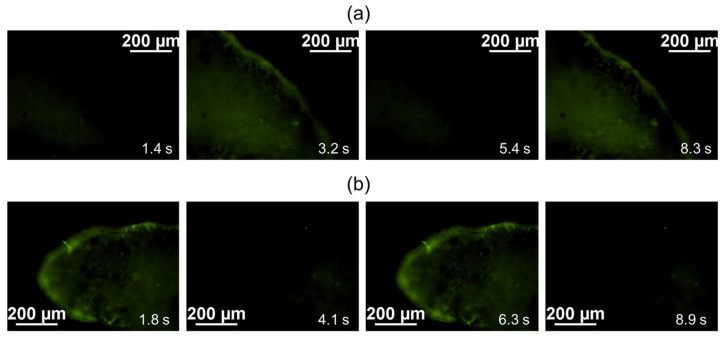
Time-lapse image of the oscillation of calcium fluxes; intracellular calcium was stained by green fluorescent calcium binding dye Fluo-8: (**a**) iPSC-derived cardiomyocyte cell layers cultured 42 days in vitro; (**b**) iPSC-derived cardiomyocyte cell layers cultured 42 days in vivo under the kidney fibrous capsule. 130 fps. Bar 200 µm.

During the analysis of calcium ion fluxes in both the samples of cardiomyocyte cell layers cultivated in vivo under the SCID mouse kidney capsule and the control samples (formed cardiomyocyte cell layers cultured in vitro in a matrigel layer at 37 °C and 5% CO_2_), a consistent and spontaneous fluctuation in Fluo-8 luminescence was observed, as depicted in Figure 3a,b, respectively.

Cardiomyocytes undergo membrane depolarization during an action potential, leading to contraction. This depolarization is triggered by the rapid influx of calcium through voltage-gated calcium channels. To visualize the calcium currents, Fluo-8 dye was used, which is commonly employed to measure action potentials and the contraction rate of cardiomyocytes [26,27]. Fluo-8 dye binds to intracellular calcium, resulting in an increase in fluorescence, thus allowing the visualization of calcium dynamics. During the repolarization phase of the action potential, as calcium is pumped out of the cell, the fluorescence of the indicator decreases, reflecting the reduction in intracellular calcium concentration [28,29].

**Figure 4:**
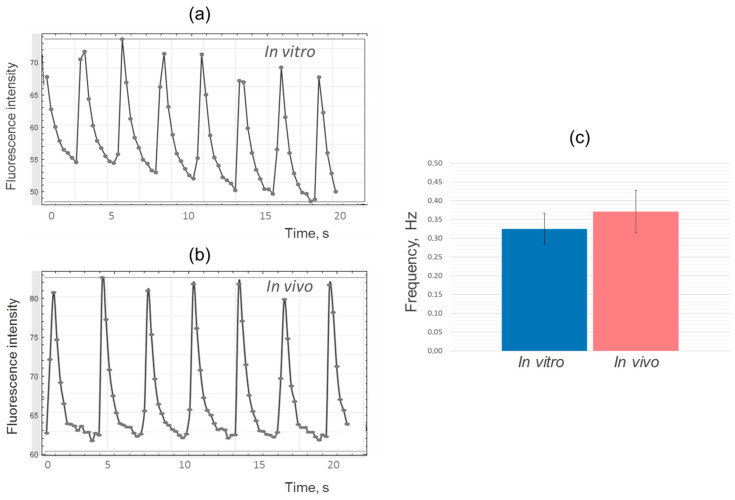
Changes in calcium binding dye fluorescence intensity versus time: (**a**) iPSC-derived cardiomyocyte cell layers cultured 42 days in vitro; (**b**) iPSC-derived cardiomyocyte cell layers cultured 42 days in vivo under the kidney fibrous capsule; (**c**) Comparison of the contraction frequency of experimental (in vivo) and control (in vitro) iPSC-derived cardiomyocyte cell layer samples.

Following the analysis of the results using the ImageJ program, fluorescence intensity change diagrams were generated for both the experimental and control samples (Figure 4a,b). The analysis revealed similarities in the shape and frequency of the peaks, indicating comparable dynamics of calcium ion fluxes and contraction frequency in iPSC-derived cardiomyocytes during the cultivation of cell layers in vitro and in vivo (Figure 4c).

It is important to note that the frequency of contractions in iPSC-derived cardiomyocytes, both in vitro and in vivo, was significantly lower compared to the frequency of contractions in adult cardiomyocytes found in the heart, which is typically around 1–1.5 Hz (60–90 times per minute) [30]. In adult cardiomyocytes, the calcium handling system is mediated by the specific spatial organization of the cellular structure, where L-type calcium channels are closely associated with ryanodine receptors on the sarcoplasmic reticulum, forming an efficient hub for excitation-contraction coupling throughout the length and width of the cell [3]. In contrast, iPSC-derived cardiomyocytes lack T-tubules (membrane organelles that extend into the interior of the myocyte) and have underdeveloped sarcoplasmic reticulum [31]. As a result, iPSC-derived cardiomyocytes rely mainly on L-type calcium channels for calcium influx, leading to slower excitation–contraction coupling [3].

**Figure 5:**
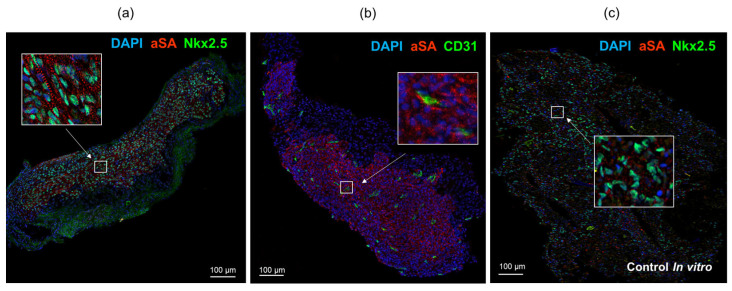
Immunofluorescence staining of iPSC-derived cardiomyocyte cell layers dissected from under the kidney capsule at day 42 (**a**,**b**) and control cell layer cultured 42 days in vitro (**c**). aSA–human sarcomeric alpha actinin (red), human Nkx2.5 (green), mouse CD31 (green). Nuclei stained with DAPI (blue). Bar 100 µm.

Immunofluorescence staining of cardiomyocyte layers using antibodies against sarcomeric actinin and cardiomyocyte transcription factor Nkx2.5 revealed the presence of human cardiomyocytes that remained in SCID mice throughout the duration of the experiment (Figure 5a). The tissue also exhibited infiltration of CD31+ capillaries of mouse origin (Figure 5b). The extracted transplants displayed elongated cell shapes and an organized sarcomere structure resembling that of cardiac myocytes within the myocardium. In contrast, the control samples, which were cultured in vitro, showed even distribution of iPSC-derived cardiomyocytes with a rounded morphology and a more disorganized arrangement of sarcomeric actinin in the cytoplasm (Figure 5c).

**Figure 6:**
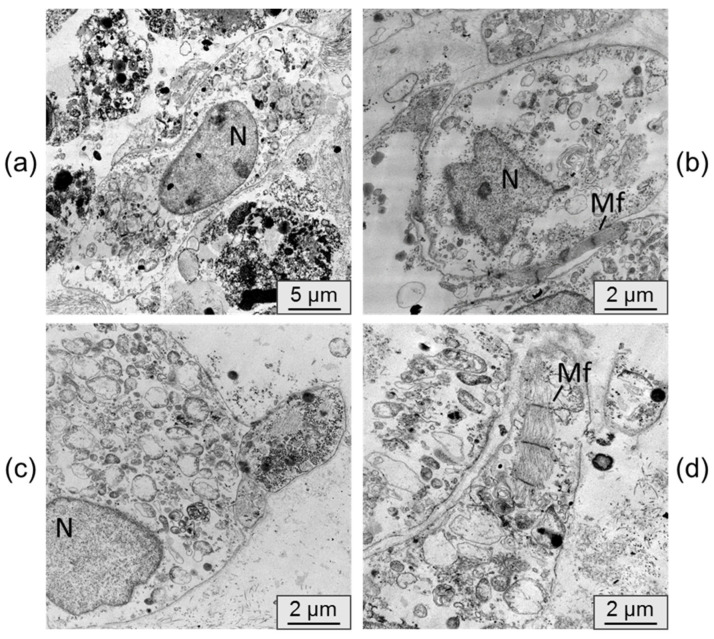
(**a**–**d**) representative transmission electron microscopy pictures of iPSC-derived cardiomyocyte cell layers cultured for 42 days in vitro. N–nucleus, Mf–myofibrils. Bar 5 µm (**a**), 2 µm (**b**–**d**).

Transmission electron microscopy was utilized to examine the alterations in cardiomyocyte ultrastructure following their persistence in an immunodeficient mouse. Electron micrographs of control samples, which consisted of cardiomyocyte cell layers cultured in vitro, are depicted in Figure 6.

**Figure 7:**
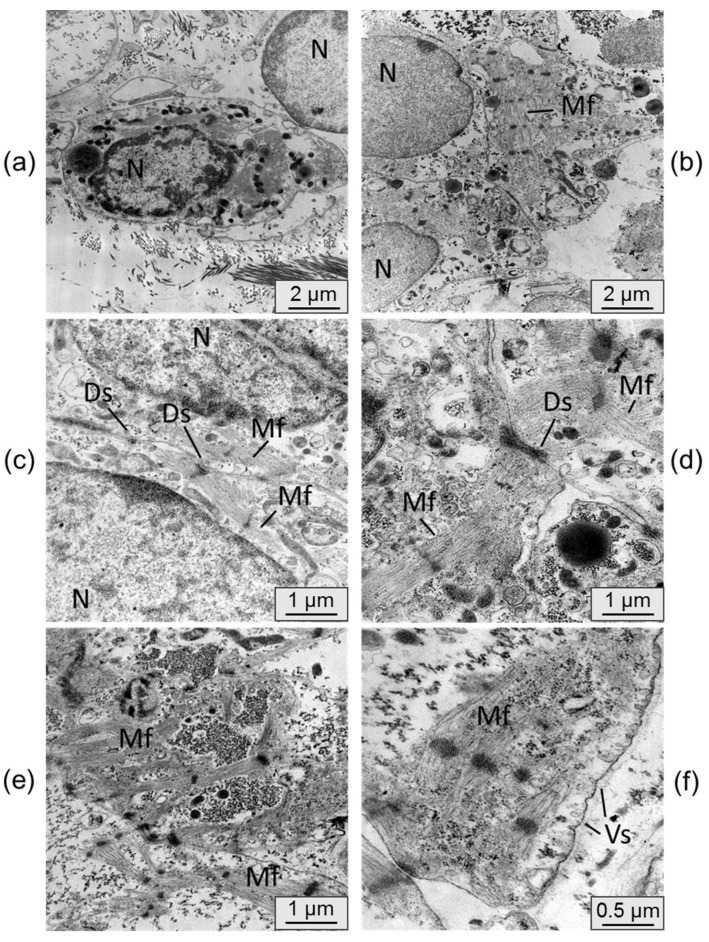
(**a**–**f**) representative transmission electron microscopy pictures of iPSC-derived cardiomyocyte cell layers cultured for 42 days in vivo under the mouse kidney capsule. N–nucleus, Mf–myofibrils, Ds–desmosomes, Vs–vesicles. Bar 2 µm (**a,b**), 1 µm (**c**–**e**), 0.5 µm (**f**).

Figure 7 displays electron micrographs of samples from cardiomyocyte cell layers that were cultured in vivo under the kidney capsule.

In the control samples, the cardiomyocytes generally lacked distinct phenotypic features of mature cardiomyocytes. These cells were typically round in shape with a nucleus containing uncondensed chromatin. The formation of sarcomeric myofibrils was observed only in isolated instances, and the organization of fibers within them was loose. The cardiomyocytes were scattered individually without forming functional connections.

The ultrastructure of the cardiomyocytes in the cell layers extracted from under the kidney capsule exhibited significant differences. The cells were densely packed, and the membranes of individual cardiomyocytes displayed mutual invaginations connected by desmosomes. The cell nuclei exhibited denser chromatin condensation. Moreover, the cells displayed a higher number of transversely striated myofibrils with a well-organized sarcomeric structure and ordered myofilaments. In some samples, there was a tendency for parallel alignment of myofibrils within the cells. Active formation of cytoplasmic vesicles was also observed in the cells cultured in vivo, whereas they were nearly absent in the control samples. These characteristics indicate a process of cell maturation and the initiation of the transition from a fetal phenotype to a mature cardiomyocyte phenotype [6].

Previous studies have demonstrated that human iPSC-derived cardiomyocytes can survive after subcutaneous administration of a Matrigel-based cell suspension to SCID mice, but only a subset of cells retain the ability to spontaneously oscillate calcium ions [32]. In this study, we have shown that when cardiomyocytes are transplanted as a functional cell layer under the fibrous capsule of the SCID mouse kidney, they maintain coordinated contraction for at least 6 weeks. Furthermore, it was observed that during their persistence in vivo, iPSC-derived cardiomyocytes develop a more organized sarcomere structure in the contractile apparatus compared to control samples cultured in vitro. This suggests that transplantation under the kidney fibrous capsule has a positive effect on the structural characteristics of the injected cells, as it partially recreates the natural microenvironment of maturing cardiomyocytes within a living organism.

Several studies have highlighted the influence of the microenvironment on the maturation of iPSC-derived cardiomyocytes. For instance, the presence of cardiac fibroblasts and endothelial cells in mixed 3D-cell culture has been shown to promote electrical and mechanical maturation of iPSC-derived cardiomyocytes through cellular interactions mediated by gap junctions [10]. Other mechanisms, such as cell–extracellular matrix interactions and paracrine effects, may also contribute to cell maturation. For example, mesenchymal stromal cells secrete various factors (VEGF, bFGF, SDF-1, and GM-CSF) that mediate differentiation and electrical coupling of iPSC-derived cardiomyocytes [33], while endothelial cells express specific extracellular matrices (ECMs; collagens I and III, fibronectin, thrombospondin-4) that enhance the sarcomere length of iPSC-derived cardiomyocytes [34]. Additionally, the addition of collagen fibril substrates to cardiomyocyte culture has been found to promote cell elongation and accelerate the development of the contractile apparatus [35]. Transplantation into the rat heart has also been shown to accelerate the maturation rate of iPSC-derived cardiomyocytes in a non-cellular autonomic manner [36].

It is worth noting that although intramyocardial transplantation has a positive impact on cardiomyocyte maturation, it may present challenges for the subsequent in vivo assessment of the functional activity of the injected cells.

## 3. Materials and Methods

### 3.1. Generation of Cardiomyocytes

A human iPSC (iMA-1L cell line, ICG SB RAS, Novosibirsk, Russia) was utilized for directed differentiation into cardiomyocytes [17]. The iPSCs were cultured on an LDEV-Free Matrigel™ hESC-qualified matrix (Corning Inc., Corning, NY, USA) in Essential-8 medium (Thermo Fisher Scientific, Waltham, MA, USA). The differentiation process followed previously published protocols that involved activating the WNT pathway using CHIR99021 (StemRD, Burlingame, CA, USA) for 48 h, followed by inhibition with IWP2 (Merck KGaA, Darmstadt, Germany) in RPMI-1640 (Lonza, Köln, Germany) with B27 supplement (Thermo Fisher Scientific, Waltham, MA, USA) without insulin [18,19].

Between days 14 and 18 of differentiation, the cells were dissociated using TrypLE Express (Thermo Fisher Scientific, Waltham, MA, USA) and transferred into Matrigel™-coated 6-well plates in RPMI-1640 medium supplemented with 20% embryonic bovine serum (Autogene Bioclear, Calne, UK) and 10 µM Y-27632 supplement (StemRD, Burlingame, CA, USA). Two days after the transfer and within one week, metabolic cell selection was conducted to purify the population of cardiomyocytes [37]. The metabolic selection medium consisted of RPMI-1640 without D-glucose (Thermo Fisher Scientific, Waltham, MA, USA), 213 μg/mL L-ascorbic acid 2-phosphate (Sigma-Aldrich, Burlington, MA, USA), 500 µg/mL recombinant human albumin expressed in *Oryza sativa* (Sigma-Aldrich, Burlington, MA, USA), and 5 mM DL-sodium lactate L4263 (Sigma-Aldrich, Burlington, MA, USA).

To form cell layers, cardiomyocytes were seeded at a density of 250,000 cells per 1 cm^2^ on temperature-sensitive Nunc UpCell plates (Thermo Fisher Scientific, Waltham, MA, USA). Once the temperature dropped to 20 °C, the cardiomyocyte cell layers were detached from the plastic and used for transplantation.

### 3.2. Flow Cytometry

The cell cultures were dissociated using Dispase (1 mg/mL) (Stemcell Technologies, Vancouver, BC, Canada) and then labeled with SIRPa-PE antibodies (#323806, Biolegend, San Diego, CA, USA) at the recommended concentrations. The stained cells were analyzed using the FACS Canto II flow cytometer (BD Biosciences, San Jose, CA, USA) with BD FACSDiva^TM^ software, version 8.0 (BD Biosciences, San Jose, CA, USA).

### 3.3. Renal Subcapsular Transplantation

The animal tests were conducted in a specific pathogen-free (SPF) vivarium at ICG SB RAS in Novosibirsk, Russia. All experiments were carried out in accordance with the protocols and recommendations for the appropriate use and care of laboratory animals as outlined in the ECC Directive 86/609/EEC. The experimental protocol was approved by the ethical board of the Institute of Cytology and Genetics (permit No. 22.4, dated 30 May 2014).

Male SCID mice (n = 10) aged 6–8 weeks and weighing 26–30 g were utilized in the study. The mice were housed in groups of 2–5 animals, consisting of siblings, in OptiMice IVC cages (Animal Care Systems, Centennial, CO, USA) at the Center for Genetic Resources of Laboratory Animals at the Institute of Cytology and Genetics (RFMEFI62119 × 0023). They had ad libitum access to water and granulated forage (Sniff, Soest, Germany). The animals were maintained in a controlled environment with a 14 h light cycle (2 a.m. to 4 p.m.) and a 10 h dark cycle (4 p.m. to 2 a.m.). The temperature was maintained at 22–24 °C, and the relative humidity was kept between 40% and 50%. The mice had unlimited access to water and food (Mucedola, Settimo Milanese, Italy).

The mice were anesthetized with Zoletil-100 (30 mg/kg) (Virbac Sante Animale, Carros, France) and Domitor (0.25 mg/kg) (Orionpharma, Espoo, Finland). The cardiomyocyte layers obtained were then transplanted under the fibrous capsule of the left kidney. Access to the kidney was gained through a left lateral subcostal incision, followed by manual eventration. Using a 200 µL tip (Axigen, Corning Inc., Corning, NY, USA), the integrity of the fibrous capsule was disrupted, and the cardiomyocyte implant was injected using an automatic pipette. After 42 days, the animals were euthanized by continuous exposure to CO_2_. The implants were extracted and divided into two parts for microscopic studies and functional tests. Cardiomyocyte cell layers that were cultured in a Matrigel™ layer (Corning Inc., Corning, NY, USA) at 37 °C and a 5% CO_2_ atmosphere throughout the experiment were used as a control.

### 3.4. Calcium Flux Assay

The formed cardiomyocyte cell layers were incubated for 30 min in a culture medium at 37 °C with a green fluorescent calcium binding dye, Fluo-8 AM (Abcam, Cambridge, UK), at a concentration of 4 μg/mL. Subsequently, the dye solution was replaced with Tyrode’s solution (Sigma-Aldrich, Burlington, MA, USA), and the oscillation of calcium fluxes in the cells was observed using an Eclipse Ti-E fluorescence microscope (Nikon, Tokyo, Japan) equipped with a DS Qi1Mc camera (Nikon, Tokyo, Japan) and the Nikon Imaging Software (NIS)–Elements Advanced Research software, version 4.30.00 (Laboratory Imaging Ltd., Praha, Czech Republic). A video with fixed camera parameters (130 fps) was recorded and processed using ImageJ software, version 1.53k (U.S. National Institutes of Health, Bethesda, MD, USA). The signal intensity versus time was plotted.

### 3.5. Preparation of Cryosections and Immunofluorescent Staining

The cardiomyocyte cell layers were frozen in Tissue-Tek OCT medium (Sakura Finetek, Tokyo, Japan) at a temperature of −22 °C. Ten-micrometer sections were obtained using a Microm HM-550 cryostat (Thermo Fisher Scientific, Waltham, MA, USA) and mounted onto SuperFrost Plus slides (Menzel-Gläser, Thermo Fisher Scientific, Waltham, MA, USA). The cryosections on slides were fixed with 4% paraformaldehyde in phosphate-buffered saline (PBS) (Sigma-Aldrich, Burlington, MA, USA) for 10 min, followed by three washes of 5 min each with PBS. Permeabilization was performed using 0.05% Triton X-100 in PBS (Sigma-Aldrich, Burlington, MA, USA) for 10 min, followed by three washes of 5 min each with PBS. The slides were then blocked with 1% bovine serum albumin (BSA) in PBS (Sigma-Aldrich, Burlington, MA, USA) for 30 min.

For immunostaining, the slides were incubated with primary antibodies overnight at 4 °C in a humidified chamber, followed by three washes of 10 min each with PBS. Next, the slides were incubated with secondary antibodies in the dark at room temperature for 1 h and washed three times for 10 min each with PBS. Nuclei were stained with Vectashield Antifade Mounting Medium with DAPI (Vector Laboratories, Newark, CA, USA). The stained samples were analyzed using an Eclipse Ti-E fluorescence microscope (Nikon, Tokyo, Japan) with the NIS–Elements Advanced Research software, version 4.30.00 (Laboratory Imaging Ltd., Praha, Czech Republic) and THUNDER Imager Live Cell & 3D Cell Culture & 3D Assay (Leica Microsystems, Wetzlar, Germany) at the Common Facilities of Microscopic Analysis of Biological Objects (ICG SB RAS, Novosibirsk, Russia).

In the immunostaining process, the following primary antibodies were used at a dilution of 1:100 with 1% BSA in PBS: Anti-human Sarcomeric Alpha Actinin antibody (#ab9465, Abcam, Cambridge, UK), Anti-human Nkx-2.5 antibody (#sc-14033, Santa Cruz Biotechnology, Santa Cruz, CA, USA), Anti-mouse CD31 antibody (#102502, Biolegend, San Diego, CA, USA). For the secondary antibody staining, the following antibodies were used at a dilution of 1:400 in 1% BSA in PBS: Alexa Fluor 488 goat anti-rabbit IgG H + L (#A11008, Life Technologies, Carlsbad, CA, USA), Alexa Fluor 488 goat anti-rat IgG H + L (#A11006, Life Technologies, Carlsbad, CA, USA), Alexa Fluor 568 goat anti-mouse IgG1 (#A21124, Life Technologies, Carlsbad, CA, USA).

These antibodies were used to visualize specific protein markers and cellular components in the cardiomyocyte cell layers.

### 3.6. Transmission Electron Microscopy

Fragments of cardiomyocyte cell layers were fixed in a 4% paraformaldehyde solution in Hanks’ medium. Subsequently, they were postfixed in a 1% OsO_4_ solution in PBS at pH 7.4 for 1 h. The samples were dehydrated using an ethanol series and then embedded in Epon ribbon (Thermo Fisher Scientific, Waltham, MA, USA). Semi-thin sections with a thickness of 1 µm were obtained using a Leica EM UC7 ultramicrotome (Leica Microsystems, Wetzlar, Germany). These sections were stained with toluidine blue and examined using a Leica DME light microscope (Leica Microsystems, Wetzlar, Germany) for preexamination. Ultra-thin sections, ranging from 70 to 100 nm in thickness, were cut using the Leica EM UC7 ultramicrotome (Leica Microsystems, Wetzlar, Germany). The sections were contrasted with a saturated aqueous solution of uranyl acetate and lead citrate. Digital photographs were captured using a JEM 1400 electron microscope (JEOL, Tokyo, Japan) at the Common Facilities of Microscopic Analysis of Biological Objects (ICG SB RAS, Novosibirsk, Russia).

### 3.7. Statistical Analysis

The statistical analysis of the results was performed using the STATISTICA 8.0 software (StatSoft Inc., Tulsa, OK, USA). The normality of data distribution was evaluated using the Shapiro–Wilk test. To determine differences between the groups, Student’s two-sample *t*-test was employed. Statistical significance was defined as *p* < 0.05. The data were presented as means ± standard deviation (SD).

## 4. Conclusions

In conclusion, our study has demonstrated that the formation of functional layers of iPSC-derived cardiomyocytes promotes accelerated cell maturation and the retention of rhythmic contraction ability after transplantation. However, at this stage of the study, we cannot definitively determine whether the generation of action potentials in the transplanted cells is due to stochastic processes in immature contractile cardiomyocytes or the activity of emerging pacemaker cells through a membrane mechanism. Further research and longer follow-up periods are necessary to draw definitive conclusions regarding the benefits of cardiomyocyte transplantation as part of a formed cell layer.

To gain a clearer understanding of the processes occurring within grafts surrounded by tissues in a living organism, we plan to conduct in vivo visualization of the formation and distribution of calcium currents. Additionally, we aim to study the response of transplanted cells to the systemic effects of neurotransmitters from the sympathetic and parasympathetic systems. These investigations will provide valuable insights into the functionality and integration of transplanted cardiomyocytes within the host tissue.

## Data Availability

Not applicable.
